# Liquid metal biomaterials: translational medicines, challenges and perspectives

**DOI:** 10.1093/nsr/nwad302

**Published:** 2023-11-29

**Authors:** Hanchi Xu, Jincheng Lu, Yikuang Xi, Xuelin Wang, Jing Liu

**Affiliations:** Department of Biomedical Engineering, School of Medicine, Tsinghua University, Beijing100084,China; Beijing Tsinghua Changgung Hospital, School of Clinical Medicine, Tsinghua University, Beijing102218, China; Department of Biomedical Engineering, School of Medicine, Tsinghua University, Beijing100084,China; Beijing Tsinghua Changgung Hospital, School of Clinical Medicine, Tsinghua University, Beijing102218, China; Shanghai World Foreign Language Academy, Shanghai200233, China; Beijing Advanced Innovation Center for Biomedical Engineering, School of Engineering Medicine, Beihang University, Beijing100191, China; Department of Biomedical Engineering, School of Medicine, Tsinghua University, Beijing100084,China; Beijing Key Lab of Cryo-Biomedical Engineering and Key Lab of Cryogenics, Technical Institute of Physics and Chemistry, Chinese Academy of Sciences, Beijing100190, China

**Keywords:** liquid metal biomaterials, translational medicine, clinical device, healthcare, therapeutics

## Abstract

Until now, significant healthcare challenges and growing urgent clinical requirements remain incompletely addressed by presently available biomedical materials. This is due to their inadequate mechanical compatibility, suboptimal physical and chemical properties, susceptibility to immune rejection, and concerns about long-term biological safety. As an alternative, liquid metal (LM) opens up a promising class of biomaterials with unique advantages like biocompatibility, flexibility, excellent electrical conductivity, and ease of functionalization. However, despite the unique advantages and successful explorations of LM in biomedical fields, widespread clinical translations and applications of LM-based medical products remain limited. This article summarizes the current status and future prospects of LM biomaterials, interprets their applications in healthcare, medical imaging, bone repair, nerve interface, and tumor therapy, etc. Opportunities to translate LM materials into medicine and obstacles encountered in practices are discussed. Following that, we outline a blueprint for LM clinics, emphasizing their potential in making new-generation artificial organs. Last, the core challenges of LM biomaterials in clinical translation, including bio-safety, material stability, and ethical concerns are also discussed. Overall, the current progress, translational medicine bottlenecks, and perspectives of LM biomaterials signify their immense potential to drive future medical breakthroughs and thus open up novel avenues for upcoming clinical practices.

## Teaser

This review highlights the diverse bioapplications of liquid metals in monitoring and imaging, tissue repairing, and tumor therapy, analyzes the translational obstacles, and envisions the future development towards artificial organs.

## INTRODUCTION

Biomaterials stand at the frontier of revolutionary transformations in clinical medicine, holding paramount significance in shaping contemporary diagnostic and therapeutic modalities [1]. Their intrinsic attributes, such as superior biocompatibility, optimal mechanical support, and tailored biodegradability, render them indispensable in advancing patient care. By seamlessly integrating with biological systems, these biomaterials have paved the way for innovations of implants, biomarkers, medical devices, biosensors, artificial organs, and sophisticated drug delivery platforms in diverse clinical settings. Moreover, a testament to their pervasive clinical applications is the widespread deployment of metal biomaterials such as titanium alloys, stainless steel, and cobalt-chromium alloys [2]. Their incorporation in fields like dentistry, orthopedics, and cardiac repair emanates from their exemplary mechanical robustness, anti-corrosion attributes, adaptability, and conductance. Besides, the potential of biomaterials as vehicles for therapeutic agents is groundbreaking [3]. They facilitate innovations like high-efficacy energy conversion substances, precision-targeted drug delivery systems and nano-drugs, aiming to enhance therapy by minimizing damage to healthy tissues and reducing side effects. Overall, with the rapid development of material science, a plethora of effective and reliable clinical biomaterials now cater to a myriad of diseases. However, many bottlenecks still exist that prevent the further spread of biomaterials for clinical applications, such as mechanical mismatch between biomaterials and human tissues, diminished function or ineffectiveness due to prolonged *in vivo* degradation, and salient biosafety concerns such as immune reactions, allergies, toxicity, and chronic inflammation [4]. Therefore, researchers urgently need to develop more innovative biomaterials to address challenges in clinical practice, ensuring long-term functional stability and reliability, including mechanical compatibility, performance stability, and long-term biological security, etc.

In recent years, the burgeoning domain of LM has piqued the intellectual curiosity of the global research community, underscored by its unparalleled and distinct properties [5]. LM, true to its nomenclature, is characterized by its ability to remain in a fluid state at ambient temperatures. These predominantly encompass sodium-potassium alloys, mercury, cesium, gallium, bismuth, and their composite alloys. However, the intrinsic challenges posed by the biological toxicity of mercury, the radioactivity of cesium, and the potent chemical activity of sodium-potassium alloys limit their potential clinical application [6]. Different from these conventional LMs, scientists have recently discovered and fabricated a series of LM alloys anchored on gallium (Ga) and bismuth (Bi) bases [5]. These alloys emerge with a suite of remarkable characteristics of low-melting point, superior electro-/thermo-conductivity, benign biocompatibility, facile modification, and rigid-soft transition, coupled with high density. These unique properties make LM considered a class of revolutionary and innovative materials, drawing interest from researchers across various disciplines, such as energy (thermal management, heat dissipation), flexible electronics (soft sensors, wearables), protection (electromagnetic interference shielding, radiation protection equipment), communications (near-field communication coil), soft robotics (transformable machines, biomimetic soft robotics), catalysis and other fields. Several reviews have been published recently focusing on LM, predominantly highlighting their material properties, design, and applications across diverse fields [7,8].

**Figure 1.**
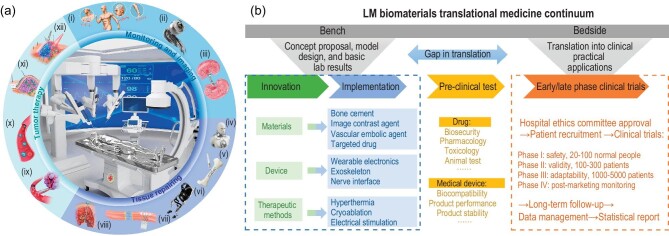
The clinical applications and translational medicine continuum of LM biomaterials. (a) Clinical applications of LM biomaterials in three fields: monitoring and imaging, tissue repairing, and tumor therapy. Monitoring and imaging applications include (i) LM healthcare monitoring devices (ii) LM biomimetic eyes (iii) LM intravascular contrast agents; Tissue repairing applications include (iv) LM external fixators (v) LM bone cement (vi) LM exoskeleton (vii) LM nerve interface (viii) LM deep brain stimulation; Tumor therapy applications include: (ix) LM vascular embolic agent (x) LM-based targeted drug delivery (xi) LM skin patch (xii) LM-enabled hybrid hyperthermia and cryoablation therapy. (b) Translational medicine continuum of LM biomaterials including innovation, implementation, pre-clinical test and early/late phase clinical trials.

Amid escalating health challenges and the advancing frontier of disease research, LM is emerging as a pivotal tool, revealing profound implications for multifaceted clinical interventions. The unique properties exhibited by LM have positioned them as valuable assets in diverse clinical applications, encompassing health monitoring, medical imaging, tissue repair, and cancer treatment (Fig. 1a). This article uniquely emphasizes LM as an emerging biomaterial with promising biomedical and clinical applications. Yet, a pronounced gap persists in the ‘bench to bedside’ process of LM biomaterials, presenting clinical translation challenges we must confront and overcome. Rather than solely focusing on laboratory studies, we undertake a more comprehensive approach, aligning clinical needs with ongoing research to highlight LM progress and challenges in real-world clinical settings. Stemming from this perspective, we innovatively delineate a translational medicine framework for LM biomaterials, encompassing 4 pivotal phases: innovation, implementation, pre-clinical test and early/late phase clinical trials (Fig. 1b). This structure facilitates a thorough examination of the opportunities, potentialities, and challenges inherent in the clinical transformation of LM biomaterials. In summary, the benefits of LM during clinical translation primarily emerge in the bench phase, where the distinctive properties of LM empower researchers to develop a myriad of functional materials and devices. The field of LM is attracting increasing attention, with innovative concepts and laboratory results laying the groundwork for clinical adaptation. However, challenges predominantly arise in the bedside phase. Both medical materials and devices must meet rigorous approval criteria before initiating clinical trials. This encompasses not only stringent biosafety, stability, and functionality standards for the materials but also adherence to local laws, regulations, and ethical considerations. Moreover, to achieve widespread clinical acceptance and application, there is a necessity for clear clinical application scenarios and a streamlined, advanced large-scale preparation technology. Addressing these challenges is crucial for the broader acceptance of LM by both clinicians and patients.

In this article, we first focus on the bio-applications of LM biomaterials according to laboratory results from the perspective of common clinical departments, and subsequently analyze the clinical translation challenge faced by LM biomaterials. Meanwhile, through evaluating the recent research of LM in the fields of healthcare monitoring, medical imaging, bone repairing, nerve connection, and tumor treatment, we interpret the application opportunities and possibilities of LM in clinical areas. Finally, we outline the perspective and discuss the challenges in developing future LM clinical medicine. Summarily, this article is dedicated to innovatively combining clinical developments and demands with research on LM bio-applications, considers the obstacles in clinical translation, and accordingly puts forward perspective suggestions for the coming smooth promotion of clinical utilization.

## HEALTHCARE

### Wearable electronics

**Figure 2.**
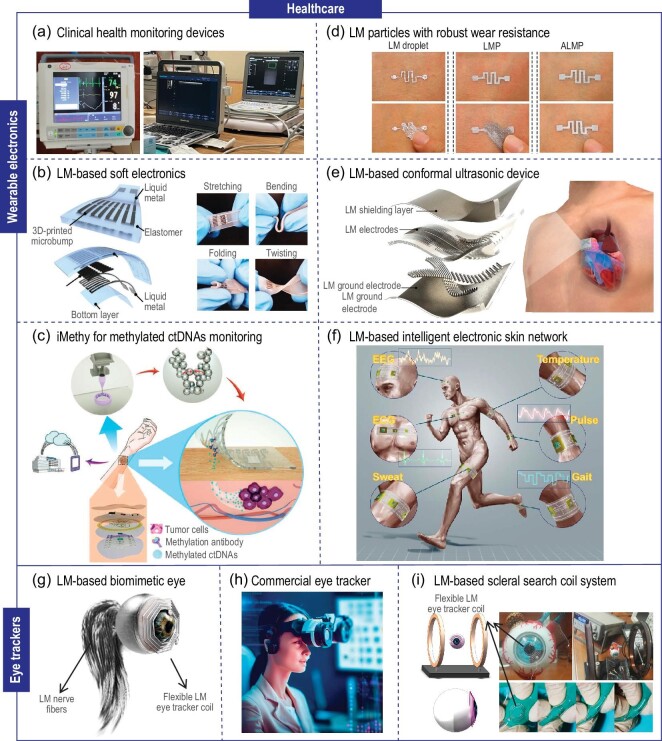
LM wearable electronics for healthcare. (a) Clinical monitoring devices: ECG monitors and ultrasound, pictures from Tsinghua Changgung Hospital. (b) Schematic view of the 3D-printed LM-based pressure sensor and its flexible tests [11]. Copyright 2020 John Wiley & Sons. (c) Schematic design of the LM-based wearable and self-healing electronic device (iMethy) for monitoring of methylated circulating tumor DNAs (ctDNAs) [17]. Copyright 2022 John Wiley & Sons. (d) LM particles with robust wear resistance for skin electronics [19]. Copyright 2022 American Chemical Society. (e) LM electrode for wearable ultrasonic device [12]. Copyright 2023 Springer Nature. (f) The intelligent electronic skin network based on the LM [20]. Copyright 2018 Springer. (g) Biomimetic eye based on LM, modified from [25]. (h) Schematic diagram of commercial eye tracker. (i) LM coil embedded in PDMS on the artificial eyeball, and adhered to a water-filled balloon to show flexibility [27]. Copyright 2018 IEEE.

Accurate monitoring of physiological parameters like heart rate, body temperature, and blood pressure is vital for preventing, diagnosing, and treating chronic diseases such as diabetes, heart disease, and hypertension. Currently, various clinical devices have been developed to monitor these physiological indicators, including sphygmomanometer electrocardiographs, and ultrasound machines (Fig. 2a). Nevertheless, these conventional devices are often large and cannot provide continuous monitoring, posing challenges for effective and long-term physiological assessment. To overcome these limitations, researchers have introduced the concept of portable and wearable electronic devices, utilizing flexible substrates (e.g. polymers) [9]. These electronic devices are designed with favorable stretchability and deformability to improve their compatibility with the human body, enabling the acquisition of superior-quality signals while avoiding mechanical damage to the person. Current materials employed in flexible electronics include organic semiconductors, conductive polymers, metal nanowires, graphene, and other 2D materials [10]. However, these often exhibit limitations in conductivity, ductility, durability, and stability. LM addresses several of these constraints: (1) Relative to the pairing of conductive metal with a flexible substrate, LM-based flexible electronics offer superior softness, maintain consistent functionality under extensive stretching, demonstrate enhanced fatigue resistance, and possess inherent self-healing and robustness; (2) In contrast to hydrogel combined with ionic conductors, LM exhibits superior electrical conductivity. Typically, Ga-based LM alloys, renowned for their excellent softness and conductivity at room temperature, have found extensive application in wearable electronics. In wearable electronics, Ga- and In-based LM compounds with metals, nonmetals, and polymers, facilitate the fabrication of diverse flexible circuits (Fig. 2b) [11], self-healing flexible electrodes [12], and stretchable packaging materials [13]. These components are crafted using techniques like microchannel infusion and 3D printing, as well as various patterning technologies, to produce a range of flexible biosensors [10]. These encompass strain sensors [14], pressure sensors [15], chemical sensors (Fig. 2c) [16,17], and other multifunctional composite sensors.

In the clinical transformation of LM wearable electronics, two critical issues need to be carefully considered. First is the affinity between the devices and skin, and the second is the mechanical compatibility between LM and packaging materials. First, to guarantee both measurement accuracy and comfort in wearable devices, affinity and permeability stand out as crucial evaluation metrics. Chen *et al*. introduced a wet-adaptive electronic skin wherein LM electrodes were embedded within a fiber mat layer, enhancing the breathability of the device [18]. Utilizing a specific bonding technique, the device can be adhered to or peeled from the skin on demand. Notably, it demonstrated exceptional adhesion exceeding 150 kPa in humid conditions, ensuring an intimate skin fit. Experimental results confirmed that prolonged adhesion does not cause skin inflammation. Employing this user-friendly electronic skin, researchers were able to monitor ECG signals and strain-induced biosensing.

Second, given the inherent fluidity of LM at ambient temperature, many researchers address potential leakage by encapsulating the device. However, introducing encapsulation materials might compromise the flexibility, functionality, and stability of LM-based wearables, primarily due to mechanical mismatches. Ding *et al*. proposed LM skin electronics with abrasion resistance without encapsulation (Fig. 2d) [19]. Skin-adhesive LM skin electronics were fabricated by integrating gallium oxide on the LM’s surface with hydroxyl groups in polyvinyl alcohol (PVA). This adherence to the skin stems from the interaction between the PVA layer and epidermal keratin, bestowing notable abrasion resistance and enabling the device to bear vertical pressures exceeding 200 N. Such encapsulation-free skin electronics adeptly circumvent constraints linked to the introduction of encapsulation materials.

LM wearable electronics have notable clinical implications, enabling measurements such as finger and joint bending angles, real-time monitoring of human pulse, body temperature, respiration, and facial expressions, capturing electrocardiograms, real-time cardiac ultrasound imaging (Fig. 2e) [12], tumor therapy, thermotherapy, and early tumor diagnostic marker monitoring [17,20–22]. These diverse applications underscore their significance in healthcare monitoring and therapeutic interventions. In short, LM-based skin electronics hold the potential for development into a comprehensive whole-body physiological signal monitoring system, consequently building an intelligent monitoring electronic skin network in the future (Fig. 2f).

### Eye trackers

As one important organ of the human body, eyes serve not only as an image-capturing organ but also as valuable indicators of an individual's psychological and physiological state, revealing information like intraocular pressure, motion frequency, and moisture. Accurate monitoring of this eye-related information is crucial for early diagnosis and effective treatment of diseases such as Parkinson's and glaucoma, offering significant value in psychology and visual sciences. Clinically, eye movements can elucidate an individual's psychological state. Many psychiatric disorders manifest with concomitant functional deficits in these movements. Consequently, eye-tracking holds promise as a predictive tool for conditions like autism and various neurological disorders [23,24]. Extensive research has been conducted in the field of eye-tracking technology and even artificial eyes (Fig. 2g) [25]. Conventional eye movement tracking techniques employ a pair of Helmholtz coils to generate a uniform magnetic field, along with two copper induction coils. According to Maxwell's equation (1):


(1) \begin{eqnarray*}
\nabla {\mathrm{\ }} \times \vec{E} = - \frac{{\partial \vec{B}}}{{\partial t}}.
\end{eqnarray*}

Changes in the magnetic flux of the induction coil caused by eye movements can be deduced from voltage amplitude and phase alterations [26]. Despite its high tracking accuracy, this system has not been widely adopted in clinical practice due to increased intraocular pressure and discomfort after wearing it. Current commercial eye-trackers typically rely on infrared or high-speed cameras to track the position of the eyeball, with drawbacks including high cost, complex equipment, and low accuracy (Fig. 2h). Consequently, an urgent need exists for technology tracking accuracy and improved comfort while wearing, thus facilitating the translation from laboratory to clinical applications. For instance, Zhao *et al*. developed a contact lens with a polydimethylsiloxane (PDMS) encapsulated Ga-In alloy metal coil as an induction coil (Fig. 2i) [27]. Theoretical and experimental results demonstrated that this LM coil exhibited outstanding measurement performance. Given that LM exhibits a fluidity akin to water at room temperature, it facilitates the integration of eye trackers with contact lenses prevalent in contemporary use. Contrary to the potential harm rigid coils might inflict on the eyeball, LM-based eye trackers enhance wearing comfort without compromising tracking precision. Actually, such LM coils can not only be considered for eye movement tracking but also for sleep monitoring, physiological signal acquisition, or even acting as artificial retinas to improve the quality of life for visually impaired patients. Furthermore, the incorporation of LM coils into contact lenses could facilitate remote control capabilities, thereby presenting a potential pathway to the realization of the metaverse.

## MEDICIAL IMAGING

Many diseases usually lack obvious symptoms in the early stages, therefore, choosing the appropriate method to image the internal tissues, organs, and lesions of the human body accurately and in real time is vital for disease prevention, diagnosis, and treatment. X-rays, computed tomography (CT), magnetic resonance imaging (MRI), and ultrasound are common medical imaging methods with the advantages of being non-invasive, fast, and accurate, and are widely used in surgery, oncology, gynecology, and other departments. The fundamental principles of various imaging methodologies rely on the differences in the absorption or reflection of light and sound signals by different tissues and organs. Consequently, enhancing absorption and reflection differences allows for the observation of improved structures. In clinical practice, high-density contrast agents are employed to increase the differential absorption of X-rays and most existing contrast agents are solutions with high-density solutes, such as iodine ion solutions [28]. However, the closed gray value under X-ray between blood and contrast solution makes the enhancement effect not ideal.

**Figure 3.**
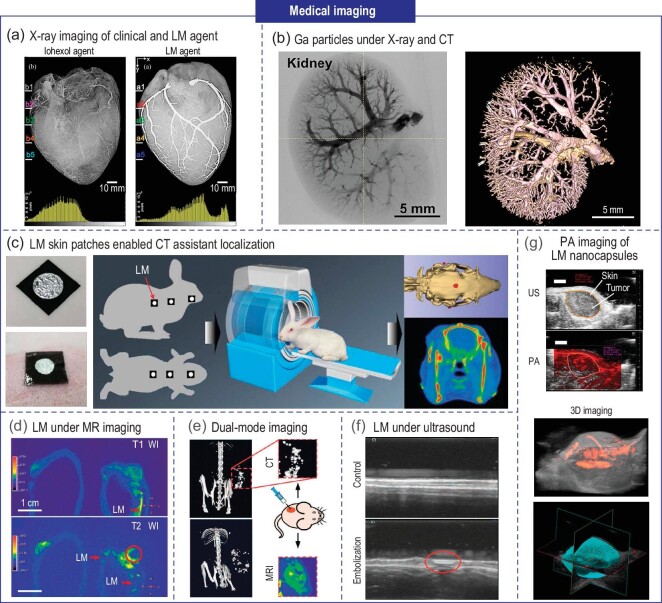
Applications of LM in the field of medical imaging. (a) Comparison of contrast effects between angiograms with LM gallium and conventional agent iohexol under X-ray irradiation [29]. Copyright 2014 IEEE. (b) Illustrated scheme of the injection process of Ga particles solution into the animal body, X-ray images and reconstructed micro-CT 3D models of rat kidney with GaPs perfused into blood vessels [30]. Copyright 2021 Elsevier. (c) The structure of CT assistant localization marker and the usage on rabbit [31]. Copyright 2020 Royal Society of Chemistry. (d) Pseudo color MRI images of T1 WI and T2 WI of the rabbit ears in embolization (right) and control (left) groups [32]. Copyright 2022 John Wiley & Sons. (e) LM particles for CT and MR dual-mode imaging [33]. Copyright 2020 John Wiley & Sons. (f) Ultrasound image of ear auricular artery with embolization (red circle: SA/LM/DOX microspheres in the artery) [35]. Copyright 2023 John Wiley & Sons. (g) US, PA and 3D imaging of tumor treated by antibody-functionalized LM nanocapsules in living mice [36]. Copyright 2017 Springer Nature.

The low melting point characteristics, high density, and biocompatibility of LM make it a promising candidate as an optimal contrast agent. For instance, Wang *et al*. innovatively introduced LM-gallium (melting point: 29.78°C) as a contrast agent, remaining liquid at body temperature, facilitating seamless and efficient injection into the target site (Fig. 3a) [29]. Due to the density difference between LM and blood, LM-filled vessels exhibit a distinct contrast, distinguishing them from surrounding tissues under X-ray imaging. Ga was injected into the vessels of porcine heart and kidney *in vitro* for contrast imaging, and the results were compared to conventional iohexol contrast agents (Fig. 3b) [30]. The experimental results strongly supported gallium's ability to improve capillary visualization and successfully reconstruct the three-dimensional structure of renal vessels via micro-CT scanning, highlighting its potential as an alternative contrast agent. In addition, the visibility of the LM under CT also allows it to be used for biopsy localization (Fig. 3c) [31].

Furthermore, researchers have investigated both the imaging efficacy and safety of LM under MR imaging (Fig. 3d) [32]. In the T1-weighted MR image, LM exhibited high signal intensity, clearly making the vessel embolized with Bi-based materials (red arrow). In the T2-weighted MR image, the clear low signal area indicated the embolic material (red arrows). (See online supplementary material for a color version of this figure.) Hence, LM holds potential for the utilization in CT-MR dual-modality imaging (Fig. 3e) [33]. In addition, under positron emission tomography (PET), Jung *et al*. achieved real-time tracking of single human breast cancer cells, which were specifically labeled with mesoporous silica nanoparticles (MSNs) carrying a high concentration of ^68^Ga radioisotope [34]. PET provided valuable insights into the dynamic processes of these cells, thus improving disease understanding and advancing clinical treatment approaches.

LM also holds promise for clinical applications in various other real-time imaging techniques, expanding its potential utility beyond the examples discussed above. For instance, Zhu *et al*. designed an eutectic GaIn (EGaIn) microsphere with a size of approximately 100 μm, which was well distinguished in ultrasound imaging (Fig. 3f) [35]. Moreover, the excellent photo-thermal conversion of LM particles has led to their application for photoacoustic (PA) imaging in living organisms (Fig. 3g) [36]. The experimental results of Chechetka *et al*. showed the highest PA signal was obtained at a wavelength of 680 nm and *in vivo* PA imaging was accomplished by injecting LM capsules into mice, indicating LM as a perfect PA contrast agent. Besides, the versatile application of LM extends beyond contrast agents, as it can fabricate flexible MR coils with ultra-stretchable polymers, enhancing patient comfort, dynamic imaging capabilities, and stable resonance frequencies, thereby advancing medical imaging technology [37]. Furthermore, LM has significant potential in radiation protection, showcasing valuable prospects in safeguarding against the harmful effects of radiation exposure [38]. Overall, LM presents versatile applications in medical imaging modalities, including X-ray, CT, MRI, ultrasound, and PA imaging, etc., enabling improved contrast and visualization for accurate diagnosis of disease.

## ORTHOPEDICS

### Bone repair

**Figure 4.**
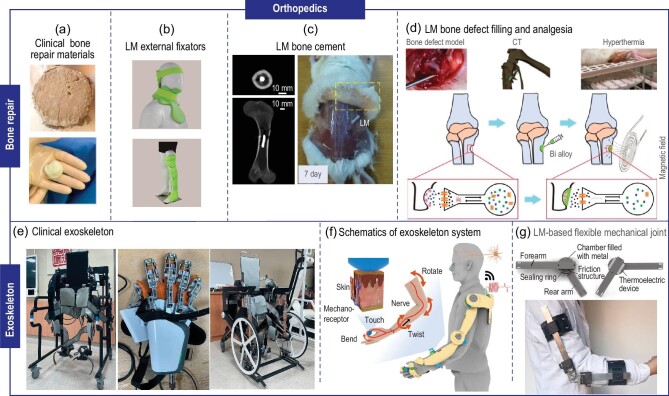
Applications of LM in orthopedics. (a) Clinical materials for bone repair: gypsum mortar (above) [39], and high-viscosity PMMA cement ready for implantation in the cavity left by debridement (below) [40]. Copyright 2022 Elsevier. (b) LM-based orthopedic external fixators, pictures from Yunnan Maiteli Medical Company. (c) The transverse and coronal sections of CT slice with LM-based bone cement inside and general observation of subcutaneous implantation of LM-based bone cement in mice after 7 days [41]. Copyright 2014 Elsevier. (d) Illustration of Bi alloy-based bone defect filling and analgesia [42]. Copyright 2021 John Wiley & Sons. (e) Exoskeleton in clinics for training people with disabilities, pictures from Beijing Tsinghua Changgung Hospital. (f) Schematics of exoskeleton system [49]. Copyright 2021 Springer Nature. (g) Prototype of the flexible mechanical joint based on a low-melting-point alloy [50]. Copyright 2014 American Society of Mechanical Engineers.

Bones serve as a vital structural element in the human body, providing support and protection, and some injured bones possess a degree of self-healing ability. In clinical practice, clinicians commonly prepare a gypsum powder-water paste to immobilize bone injuries like fractures, sprains, or joint dislocations (Fig. 4a) [39]. This paste hardens and helps promote optimal healing by limiting movement. Recently, with the intriguing nature of its low melting point, LM has emerged as an alternative in the field of external fixation. Yunnan Maiteli Company, for example, has developed LM-based orthopedic external fixators (Fig. 4b). The procedure for using LM-based orthopedic external fixators is simple. The fixator is firstly immersed in hot water to induce either the complete or partial melting of LM, allowing precise adaptation to diverse injuries. After natural cooling at room temperature, it gains high mechanical strength, providing support to injured parts. Furthermore, the removal of this fixator is notably convenient, mitigating the risk of injury from the excessive force encountered with conventional clinical plasters. Additionally, in contrast to standard plasters, LM-based orthopedic external fixators are reusable by simply replicating the aforementioned procedure, aligning with sustainable recycling principles. In cases of severe injuries necessitating surgical intervention, bone cement is crucial for filling bone defects and connecting implanted bones to tissues, effectively reducing the risk of looseness or dislocation. Commonly used bone cement is generally a non-metallic material—polymethyl methacrylate (PMMA) (Fig. 4a) [40]. In clinical practice, PMMA and calcium hydroxide (Ca(OH)_2_) are mixed and injected into the target site in order to provide support after solidification. However, PMMA bone cement has some drawbacks such as toxicity of its raw material, uncontrolled polymerization, and excessive heat generation during the reaction process. The mechanical strength, biocompatibility, and radiopacity make LM an ideal alternative. Yi *et al*. first introduced an innovative injectable bone cement with a low-melting-point alloy (35.0Bi-48.6In-16.0Sn-0.4 Zn) (Fig. 4c) [41]. It exhibited rapid solidification, minimal energy release, and reduced cytotoxicity, which are promising advancements in this field. Experimental results indicated a compressive strength of 37.6 ± 1.56 MPa for this material. Hematoxylin eosin staining revealed that the surrounding bone tissue maintained structural integrity after the injection of LM-based bone cement, with no thermal damage observed. Additionally, the inherent radiopacity of LM enabled its utilization as a contrast agent during radiographic imaging, significantly enhancing surgical guidance. Based on the above research, He *et al*. further demonstrated that the LM-based cement maintained immobility for 210 days (Fig. 4d) [42]. This obviated the necessity for additional interventions arising from material displacement, showcasing its injectability and long-term bone repair capabilities. Besides, due to the excellent thermal and electrical conductivity of LM, researchers employed an electromagnetic heater to remotely heat this alloy bone cement, effectively relieving pain deriving from bone defects. Abbasi *et al*. demonstrated that LM-based bone cement became molten at ∼60°C, allowing for its removal from the body via suction [43]. This approach circumvented the complications, pain, and risks associated with a second surgery. Moreover, *in vivo* and *in vitro* experiments further demonstrated that higher LM melting points and quantities increased thermal damage to bone tissue during treatment [44]. Therefore, LM-related parameters should be adjusted according to clinical needs, ensuring adaptation to different application scenarios while minimizing tissue damage.

Generally, promoting bone regeneration is also an essential component of the overall bone repair process, exhibiting significance in achieving successful clinical outcomes. Bone regeneration is usually a complex and coordinated process, mainly consisting of three continuous stages: the inflammatory phase, the tissue formation phase, and the tissue remodeling phase, respectively [45]. Studies have demonstrated that matrix stiffness is an essential factor influencing stem cell differentiation, with varying matrix stiffness levels being optimal for different stages of differentiation [46]. However, the current biomaterials employed in bone tissue engineering lack dynamic adaptability to varying stiffness requirements during different stages, highlighting the need for innovative materials with tunable stiffness properties. Numerous experimental studies have demonstrated the feasibility of utilizing magnetic fields as a means to modulate and manipulate the stiffness characteristics of biomaterials [47]. As a result, Li *et al*. combined magnetic silica particles (Fe@SiO_2_) with GaInSn to create magnetic LM (MLM) and applied it to bone tissue engineering [48]. The arrangement of magnetic particles varied with the strength of the magnetic field, thereby achieving dynamic stiffness regulation. As the external magnetic field increased from 0 to 0.4 T, the stiffness of MLM increased from 3.58 ± 0.48 MPa to 14.32 ± 0.97 MPa, thereby exhibiting dynamic stiffness variation when used as bone tissue engineering material. The experimental results elucidated the favorable biocompatibility of MLM scaffolds, which promoted the osteogenic differentiation of mesenchymal stem cells, thus suggesting the potential for MLM scaffolds with dynamic stiffness to augment bone regeneration and integration.

In summary, the distinctive properties of LM with room temperature solid-liquid phase transition, mechanical strength, biocompatibility, and radiopacity render it a promising and viable alternative to conventional bone repair materials for the fabrication of external fixators, bone cement, and bone scaffold, thereby greatly facilitating bone repair.

### Exoskeleton

In clinical rehabilitation, the exoskeleton is a common medical device used to assist patients with post-surgical or neurological disorders who are unable to move or dominate their limbs independently, and to facilitate the recovery of motor function (Fig. 4e). High demands are placed on the flexibility and mechanical strength of exoskeletons in order to better accommodate human movement and withstand workloads (Fig. 4f) [49]. Conventional exoskeleton joints using bearings and hydraulics for flexibility and load-bearing capacity, often suffer from complex fabrication, limited stability, and fatigue-related issues, shortening their lifespan. To solve these problems, Deng *et al*. proposed a flexible exoskeleton mechanical joint based on Bi_32.5_In_51_Sn_16.5_, with a melting point of 60°C (Fig. 4g) [50]. The solid phase bore the load, while the liquid phase ensured flexible degrees of freedom. Experiments also showed that this alloy exhibited a rapid response time, achieving a highly efficient solid-liquid phase transition. Additionally, Meerbeek *et al*. designed an elastic double-continuous foam by combining LM and an elastomer, exhibiting reversible stiffness changes, shape memory, and self-healing properties [51]. Yun *et al*. developed a self-triggered variable stiffness elastomer by filling PDMS with EGaIn and field metal, which could protect robotic hands from excessive compression, bending, and twisting [52]. Conclusively, these findings suggest that the incorporation of LM enables the development of highly functional and efficient wearable rigid-flexible exoskeletons, presenting versatile clinical applications.

## NERVE INTERFACE

### Nerve connections and function recovery

The human nervous system consists of central and peripheral nervous systems. Peripheral nerve injuries, often caused by external forces resulting in nerve rupture, can have severe consequences, including limited mobility and even lifelong disability. Clinical nerve repair techniques vary based on damage severity, including surgical neurorrhaphy for minor injury gaps and nerve grafting for extensive damage [53]. However, autologous grafting encounters obstacles like secondary surgery and limited nerve sources, while allografts face immune rejection issues. Some researchers have used natural and synthetic materials to develop artificial nerve conduits, but their application is still restricted to certain injuries [54]. In addition, numerous studies have explored growth factors for promoting peripheral nerve regeneration, but regeneration typically entails a protracted process during which surrounding muscles may undergo atrophy and related problems [55].

**Figure 5.**
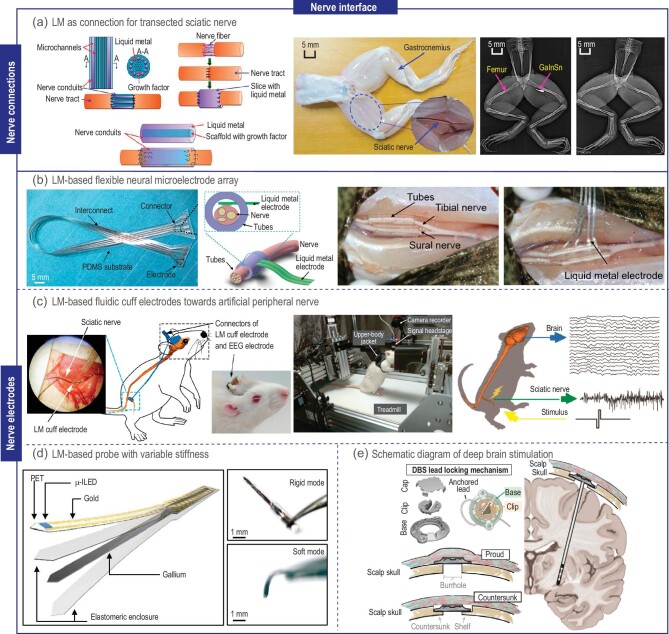
Applications of LM in nerve interface. (a) Three kinds of nerve conduits to repair the injured peripheral nerve, and the photograph and plain radiograph of bullfrog's lower body after injecting GaInSn alloy [56]. Copyright 2014 arXiv. (b) Schematic diagram of LM nerve electrode and pictures of electrodes implanted into the bullfrog sural nerve and tibial nerve [58]. Copyright 2017 Institute of Physics Publishing. (c) The positions of LM cuff electrode and EEG electrode array inside the rat body, and graphical representation of sciatic nerve signals and EEG after stimulation with bipolar pulses in freely moving rats on a treadmill [59]. Copyright 2022 Elsevier. (d) LM-based probe with variable stiffness [61]. Copyright 2019 Science. (e) Schematic diagram of deep brain stimulation device [62]. Copyright 2022 Elsevier.

Researchers have identified the exceptional conductivity and softness of LM, positioning it as a promising material for nerve repair due to its potential to facilitate the transmission of electrical signals and compatibility with the soft tissue components of nerves. Zhang *et al*. introduced for the first time a GaInSn alloy to reconnect frog sciatic nerves and convey electrical signals (Fig. 5a) [56]. By measuring the resistivity and impedance of LM, its basic electrical properties and visibility under X-ray were evaluated. Experimental results showed that the electrical signals from the reconnected sciatic nerve closely resembled those from the intact sciatic nerve, with only minor differences at the wave peaks and troughs. This indicated effective repair of transected sciatic nerves *in vitro* using GaInSn alloy. Liu *et al*. conducted analogous experiments on mouse sciatic nerves, which are closer to the human nervous system, demonstrating that mice reconnected with LM did not exhibit gastrocnemius muscle atrophy until the third month [57].

### Nerve electrode

Furthermore, for nerves experiencing functional damage without rupture, LM electrode implantation for electrical stimulation of the injured nerve could restore functionality. Guo *et al*. proposed a flexible neural microelectrode array system based on GaIn alloy encapsulated within a flexible polymer (PDMS) (Fig. 5b) [58]. Experiments showed that dead frogs implanted with flexible neural microelectrode arrays could rhythmically contract and move their lower limbs under electrical stimulation. Expanding upon this research, Tang *et al*. used LM to create a fluidic cuff electrode applied in rat (Fig. 5c) [59]. The experiment showed the LM cuff electrode possessed long-term effectiveness in nerve signal transmission, capturing high signal-to-noise sciatic nerve signals in freely moving rat. Stimulation experiments revealed that the LM electrode could convey nerve stimulation to peripheral nerves, thereby activating the somatosensory cortex in the brain.

Not only in the field of peripheral nervous systems, LM electrodes are also very effective in stimulating the central nervous system. Hallfors *et al*. successfully implanted flexible electrodes made from gallium alloys into the brain, achieving effective stimulation of neurons [60]. Besides, Byun *et al*. utilized the solid-liquid phase transition capability of LM at room temperature to fabricate a stiffness-variable neural probe (Fig. 5d) [61]. In its rigid state, the probe was inserted through the scalp and gradually softened under body temperature, adapting to brain tissue movement. This approach effectively reduced wound size and mitigated inflammatory glial reactions, making it applicable to deep brain stimulation therapy (Fig. 5e) [62].

In conclusion, these studies indicate that LM electrodes hold the potential for connecting, replacing, and even enhancing nerves, offering substantial value in the field of nerve repair. LM-based electrodes and devices exhibit promising applications in various nerve repair and regeneration scenarios. Their unique properties, including high electrical conductivity, softness, and biocompatibility, make them ideal for interfacing with the nervous system. Combining LM technology with other advancements, such as growth factors and drug delivery systems, could lead to more effective and comprehensive nerve repair strategies.

## TUMOR THERAPY

### Vascular embolic agent

**Figure 6.**
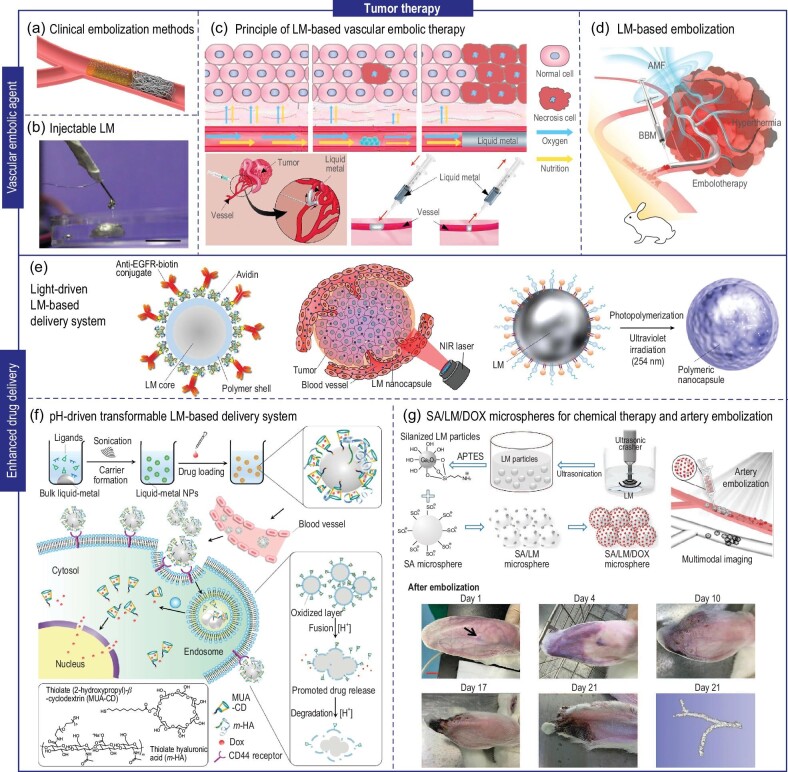
LM for tumor therapy through vascular embolization and drug delivery. (a) Schematic diagram of clinical liquid/solid vascular embolization. (b) Injectable LM [32]. Copyright 2022 John Wiley & Sons. (c) The principle illustration of LM-based tumor vascular embolization therapy [69]. Copyright 2014 arXiv. (d) The injectable liquid embolic agent enabled tumor embolization and hyperthermia [32]. Copyright 2022 John Wiley & Sons. (e) Illustration of the component of a transformable LM nanocapsule, optical control of LM nanocapsules transformation, and TEM images of laser-induced morphological changes in LM nanocapsules [36]. Copyright 2017 Springer Nature. (f) Schematic design of the pH-driven transformable LM-based delivery system [75]. Copyright 2015 Springer Nature. (g) Illustration of SA/LM/DOX microspheres for chemical therapy and artery embolization [35]. Copyright 2023 John Wiley & Sons.

Unlike normal cells, tumor cells possess an inherent ability for accelerated proliferation and metastasis, necessitating ample oxygen and nutrients to support their growth. Disrupting the nutrient supply from tumor-associated blood vessels represents a potential strategy to hinder tumor proliferation and metastasis [63]. On the one hand, scientists have endeavored to develop a variety of angiogenesis inhibitors to suppress the growth of blood vessels [64]. However, long-term drug usage leading to resistance and selectively inhibiting tumor-associated blood vessels without damaging normal vessels are challenging issues [65]. On the other hand, advancements in medical imaging and catheter techniques drive research into effective embolic agents capable of blood vessel occlusion. Currently, clinical vascular embolic agents include both solid and liquid embolic agents, offering diverse options for effective vascular occlusion. Solid embolic materials encompass both permanent solid embolic materials (such as coil springs, PVA particles, sodium alginate microspheres, etc.) and absorbable solid embolic materials (like gelatin sponge, etc.), providing plenty of choices for specific clinical requirements (Fig. 6a). However, solid embolic agents encounter challenges in precise placement and complete vessel filling, particularly in vessels with diverse and complex morphologies, leaving gaps for continued nutrient delivery. Moreover, the occlusion effect of absorbable embolic materials and the removal issues of permanent embolic materials also need to be considered in clinical settings. Liquid embolic materials like anhydrous ethanol and iodized oil, show the advantage of direct injection into tumor blood vessels, achieving complete vessel filling due to their fluidity. Nevertheless, using liquid embolic materials in tumor blood vessels faces challenges from potential washout owing to blood flow dynamics, compromising embolic effectiveness, and risking toxicity at distant body sites following metastasis [66–68].

LM, with its solid-liquid phase transition near body temperature, can serve as a potential embolic agent. It allows the complete filling of blood vessels when injected in its liquid phase, subsequently solidifying *in vivo* to effectively embolize the vessel (Fig. 6b and 6c) [32,69]. For example, Duan *et al*. employed Bi-based multifunctional LM, achieving rapid vascular occlusion at ∼40°C (Fig. 6d) [32]. Under an alternating magnetic field (AMF), the embolic agent can also generate a thermal effect due to eddy currents, enabling a synergistic treatment combining thermal therapy and embolization therapy. Wang *et al*. designed a magnetic LM nanocomposite Fe@EGaIn, achieving embolization-chemotherapy-hyperthermia tri-modal synergistic cancer therapy [70]. As discussed above, the radiopacity of LM makes it a traceable and observable embolic agent under X-rays. Due to the low melting point characteristic of LM, once the embolization task is finalized, LM can be melted through localized heating. Subsequently, it can be extracted using an injection needle, guided and positioned by imaging techniques. This approach circumvents potential toxicity and safety concerns stemming from the extended presence of the embolizing agent in the body, as well as trauma from subsequent surgical interventions.

Summarily, the inherent solid-liquid transition capability of LM confers distinct advantages as a vascular embolic agent for tumor embolization therapy, thus facilitating its practical implementation.

### Enhanced drug delivery

Chemotherapy, a widely common tumor therapy method, employs highly toxic chemical agents to eliminate cancer cells. However, chemotherapy's lack of selectivity in targeting malignant cells and the potential for drug resistance pose challenges due to the resulting damage to normal cells and limited long-term effectiveness [71,72]. Consequently, developing accurate targeting strategies to improve drug delivery specifically to tumor cells is a pressing challenge in the chemotherapy field. Small molecule kinase inhibitors and monoclonal antibodies like trastuzumab, represent clinically significant targeted therapeutics that selectively modify signaling pathways, offering effective treatment options for breast cancer [73]. Additionally, researchers have investigated vectors, including cells and nanocarriers, as effective drug delivery systems to realize targeted therapy by either passive enhanced permeability and retention effect of nanoparticles or modification of ligand sites to actively deliver drugs to tumor sites [74]. Recent research revealed that nanoscale LM particles were enveloped in a gallium oxide layer. This layer facilitates surface ligand functionalization, permitting the integration of LM particles with established drug delivery systems through surface modification. Such integration enhanced the therapeutic efficacy of drug delivery systems, optimizing synergistic therapy and controlling drug release. Most noteworthy, recent studies have highlighted the potential of LM in targeted drug delivery, leveraging their stimuli-responsive morphology alterations and ligand-functionalized surface for controlled drug release.

For example, Chechetka *et al*. demonstrated that the polymerization of EGaIn nanoparticles with functional phospholipids could improve light responsiveness (Fig. 6e) [36]. Under NIR irradiation, the shape of nanocapsules changed, thus enabling drug release. Lu *et al*. proposed an LM nanosphere composed of an EGaIn core and a thiolated polymer shell for targeted drug delivery (Fig. 6f) [75]. EGaIn nanospheres, when ultrasonically modified with thiolated-β-cyclodextrin and thiolated hyaluronic acid (HA) ligands, acquire the capability to load drugs and target tumor cells. HA specifically targets the CD44 receptor, frequently overexpressed on many tumor cell surfaces. Upon intravenous injection, doxorubicin-loaded LM nanospheres are anticipated to concentrate at the tumor site owing to both passive and active targeting mechanisms. They enter cells through endocytosis and release drugs in weakly acidic intracellular environments. Experimental results showed that using LM nanospheres for drug delivery resulted in better tumor inhibition than free doxorubicin. Moreover, researchers found that LM nanoparticles gradually degraded in tumor environments, mitigating the systemic toxicity commonly associated with non-degradable inorganic nanoparticles. Zhu *et al*. designed SA/LM microspheres with a negatively charged surface that adsorbed positively charged drugs like DOX, achieving embolization-chemotherapy synergy with a 1-month drug release duration (Fig. 6g) [35].

Leveraging the responsiveness of LM nanoparticles to external stimuli, the drug delivery system facilitates controlled drug release. This promotes precision in tumor cell treatment. Concurrently, combining synergistic treatments, like chemotherapy with thermotherapy and embolization therapy, amplifies the therapeutic outcome. Furthermore, the ligand-functionalized LM surface augments the drug delivery system's potential to integrate with other substances, thereby enhancing its application prospects. Nonetheless, the incorporation of metal particles introduces ambiguities regarding the system's safety. Consequently, comprehensive experiments are imperative to validate its safety, stability, subsequent degradability, and metabolizability.

### Hyperthermia

**Figure 7.**
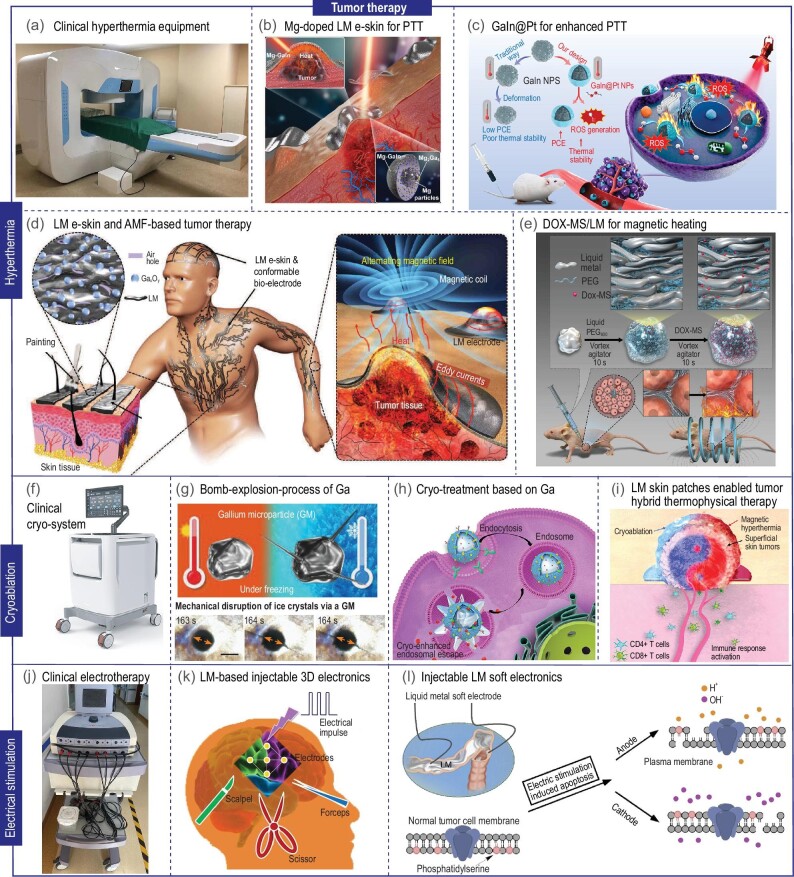
LM for tumor therapy through hyperthermia, cryoablation, and electrical stimulation, vascular embolization and drug delivery. (a) Clinical hyperthermia equipment, picture from Tsinghua Changgung Hospital. (b) Mg-doped LM for skin tumor photothermal therapy [78]. Copyright 2018 John Wiley & Sons. (c) Illustration of core-shell GaIn@Pt heterogeneous NPs for enhanced photothermal therapy [79]. Copyright 2021 Elsevier. (d) Schematic illustration of LM e-skin and AMF-based tumor therapy and pattern with O-GaIn directly printed on skin surface [81]. Copyright 2019 John Wiley & Sons. (e) Schematic of combined therapy in breast cancer with DOX-MS/LM [82]. Copyright 2019 John Wiley & Sons. (f) Clinical AI Epic Co-Ablation System, pictures from Hygea company. (g) Schematic of gallium microparticles (GMs) induced mechanical disruption of tumors for cryoablation [33]. Copyright 2020 John Wiley & Sons. (h) Illustration of cryo-facilitated LM particle transformation for endosomal escape [30]. Copyright 2021 Elsevier. (i) Flexible skin patch enabled tumor hybrid thermophysical therapy [86]. Copyright 2022 John Wiley & Sons. (j) Clinical medium frequency electrical stimulation therapy instrument, picture from Tsinghua Changgung Hospital. (k) LM-based injectable electrodes [90]. Copyright 2013 Springer Nature. (l) A schematic illustration of the conceptual application and the mechanism induced by injectable LM soft electronics in EChT and design of EChT with LM electrodes *in vitro* [91]. Copyright 2017 Elsevier.

Hyperthermia is also a common tumor therapy, with numerous evidences revealing its inhibitory effects on tumor cell activity at temperatures between 38 and 42°C, leading to apoptosis and significant tumor cell damage or death [76]. Hyperthermia raises body or tumor tissue temperature to damage tumor cells through thermal effects, offering a relatively straightforward technique with minimal side effects. It is often used as an adjunct to surgery, chemotherapy, and radiation therapy. Extracorporeal radiofrequency hyperthermia machines are commonly used in clinics, employing a non-contact treatment method that heats tissues to cytotoxic temperature (42.5–43.5°C) for 60–120 minutes by radiofrequency waves (Fig. 7a). However, this method inevitably causes unnecessary thermal damage to surrounding normal tissues, and the irregular shape of tumors complicates precise hyperthermia. LM-based targeted hyperthermia leverages the thermal conductivity, adaptability, and biocompatibility of LM as an ideal heat transfer medium for precise hyperthermia.

LM-enabled hyperthermia can be further classified into chemical, magnetic, and photothermal therapies based on different heat sources. First, chemical hyperthermia relies on the heat generated from exothermic chemical reactions. Unlike conventional thermal therapy, this method presents highly localized heating performance, which can effectively reduce thermal damage to normal tissues. Room-temperature liquid alkali metals, such as sodium (Na) and potassium (K), can be injected into tumor sites, where they react with water in the tissues to release substantial heat, damaging tumor cells. The reaction products are common sodium and potassium ions, which do not produce toxicity in the human body [77]. Second, photothermal therapy (PTT) utilizes photothermal conversion agents to convert light energy, such as near-infrared light, into heat energy. LM with excellent photothermal conversion capabilities has received widespread attention in the field of photothermal therapy (Fig. 7b) [78]. Structural stability and PTT efficiency can be improved by modifying inorganic silica, platinum, gold, etc. on the surface of LM particles (Fig. 7c) [79]. Third, magnetic hyperthermia (MH), with its tissue-penetrating capability, holds promise for treating diverse solid tumors, including deep-seated tumors. Conventional magnetothermal therapy generally utilizes magnetic nano agents but often requires a strong AMF for effective tumor treatment [80]. Wang *et al*. suggested utilizing the vortex thermal effect of nonmagnetic GaIn LM under AMF for low-intensity thermal ablation of tumors, and this effect was experimentally demonstrated in subcutaneous breast tumors and deep-seated orthotopic liver tumors in mice (Fig. 7d) [81]. Wang *et al*. studied the induction heating performance of EGaIn under external magnetic fields, achieving pH/magnetic field dual-responsive chemical-thermal synergistic therapy with DOX-MS/LM microspheres (Fig. 7e) [82]. In addition to electromagnetic waves, microwaves (MWs) also present the advantage of deep penetration. Wu *et al*. found that LM ultra particles activated by MW irradiation could generate cytotoxic reactive oxygen species (ROS), achieving chemical dynamic therapy and thermal therapy by loading LM and MW heated sensitizer ionic liquids (ILs) in mesoporous ZrO_2_ nanoparticles [83]. Summarily, LM-based hyperthermia offers precise and effective tumor therapy with highly efficient heat delivery.

### Cryoablation

Besides temperature modulation and chemotherapy, mechanical destruction is a viable tumor therapy approach, including high-intensity-focused ultrasound [84], external magnetic [85], and liquid nitrogen (Fig. 7f). Experiments showed that external stimuli can induce morphological changes in LM particles at the micro/nanoscale. For example, Sun *et al*. observed that 200 μm LM particles underwent a ‘bomb’-like explosion at low temperatures, resulting in a volumetric expansion, which mechanically damaged nearby solid chitosan ice crystals (Fig. 7g) [33]. Building on this observation, Wang *et al*. noticed that the shape of cell membrane-encapsulated Ga particles changed from spherical to cactus-like structures under low temperatures (Fig. 7h) [30]. When injected near tumor tissues, membrane-coated Ga particles loaded with paclitaxel (PTX) entered tumor cells via endocytosis. Under low-temperature stimulation, these particles experienced dramatic morphological changes, mechanically disrupting the lysosomal membrane, releasing hydrolytic enzymes, and simultaneously releasing drugs. Experimental results demonstrated that Ga/M/PPs exhibited a strong inhibitory effect on tumor growth. Furthermore, Sun *et al*. proposed that non-invasive cryoablation and thermotherapy could be achieved by LM-based flexible patches (Fig. 7i) [86]. In conclusion, incorporating LM particles at low temperatures enables mechanical destruction through external stimuli, enabling innovative tumor cryoablation approaches.

### Electrical stimulation

Clinically, electrical stimulation has been implemented in treating various diseases, including cardiac disorders, Parkinson's disease, depression, and schizophrenia (Fig. 7j) [87]. Owing to the unique softness and conductivity of LM, flexible electrodes have been considered for external or internal placement to provide electrical stimulation for tumor treatment. Li *et al*. utilized a spray-coating method to directly print LM onto the skin at the site of the tumor, forming a skin electrode, and observed significant inhibition of melanoma tumor growth in mice after 6 days of 90-minute electrical stimulation treatment, highlighting a promising potential of LM-based tumor therapy [21]. Furthermore, Ma *et al*. successfully demonstrated remotely controlled tumor thermal therapy utilizing LM skin patches [88]. After treatments, the LM on skin could be easily removed using medical alcohol. To increase the stability of LM electronic skin, Xu *et al*. proposed a phase-separated porous LM-elastomer composite, enhancing the stability of LM electronic skin by combining ductility, leak resistance, antibacterial properties, and breathability for enhanced longevity and comfort [89].

Besides transcutaneous electrical stimulation, researchers have explored implanting LM electrodes directly into the body for electrical stimulation therapy. Jin *et al*. introduced the concept of injectable electrodes, directly injecting encapsulating materials and LM into target tissues to create 3D-compliant medical electronics as needed (Fig. 7k) [90]. The fluidity of LM enabled it to adapt to tumors with complex shapes when injected. Besides, Sun *et al*. proposed an injectable electrode electrochemical treatment (EChT) modality (Fig. 7l) [91]. Upon current or voltage application, electrolytes begin to electrolyze, producing a series of toxic electrochemical byproducts for tumor treatment. Compared to traditional platinum electrodes, LM electrodes generated double the current at the same voltage, producing more electrochemical byproducts. This effectively impeded tumor growth, extended the lifespan of mice, and demonstrated superior tumor treatment efficacy.

### Anti-microbial and inflammatory

**Figure 8.**
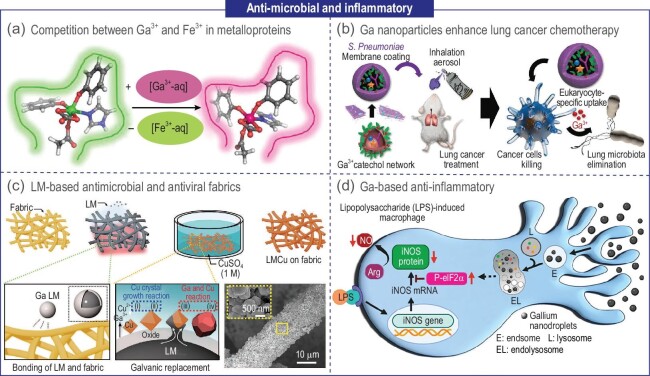
LM for anti-microbial and inflammatory. (a) Schematic of competition between Ga^3+^ and Fe^3+^ in metalloproteins [93]. Copyright 2016 American Chemical Society. (b) Ga for cancer chemotherapy [94]. Copyright 2023 John Wiley & Sons. (c) Schematic and principle of LM-based antimicrobial fabrics [95]. Copyright 2021 John Wiley & Sons. (d) Mechanism of Ga-based anti-inflammatory [96]. Copyright 2022 American Chemical Society.

In clinical settings, bacterial infections often underlie prevalent diseases like influenza and pneumonia. The advent of antibiotics ushered in a transformative era of antimicrobial therapy. Nevertheless, the excessive use of antibiotics has culminated in the rise of bacterial resistance, posing a significant challenge to the effective treatment of associated diseases. Consequently, the need for innovative antimicrobial methodologies is paramount. Studies have highlighted the antimicrobial potential of LM gallium and its ions, which exert their effects by disrupting iron metabolism, generating ROS, and causing thermal and mechanical damage [92]. Notably, the predominant theory posits the ionic competition between Ga^3+^ and Fe^3+^ (Fig. 8a) [93]. Iron ions are crucial for cellular metabolic functions. Given the structural and chemical similarities between gallium and iron ions—including ionic radius, ionization potential, and electron affinity, gallium ions can interfere with cellular metabolism by competing for protein binding sites with iron ions, achieving an antimicrobial effect.

Han *et al*. synthesized GaTa nanoparticles through the coordination of gallium ions and polyphenols (tannin, Ta) (Fig. 8b) [94]. These nanoparticles were subsequently encapsulated within a biocompatible nanofilm derived from S. Pneumoniae, culminating in the creation of the so-called Trojan horse nanoparticles: GaTa-CP@Eto NPs. Within the tumor environment, Ga^3+^ was released, targeting bacterial iron respiration. In studies involving mice with lung cancer, GaTa-CP@Eto NPs effectively curtailed tumor growth and enhanced survival rates. Beyond oncological applications, the antimicrobial attributes of gallium ions have potential in textile integration. Kwon *et al*. adeptly anchored LM-copper alloy (LMCu) particles onto fabric surfaces, capitalizing on the displacement reaction between gallium and copper ions and leveraging the inherent adhesive properties of LM on textiles (Fig. 8c) [95]. Subsequent analysis affirmed the antimicrobial efficacy of LMCu-imbued fabrics against both Gram-positive and Gram-negative bacteria, fungi, and respiratory RNA human viruses. This innovation holds promise for applications in medical and public settings, including medical masks and protective wear, potentially mitigating public health concerns.

However, the anti-inflammatory effects exhibited by Ga ions presented adverse effects by disturbing iron homeostasis. Zhang *et al*. innovatively employed gallium particles to suggest an anti-inflammatory mechanism that does not disrupt iron homeostasis (Fig. 8d) [96]. At sub-micrometer or nanometer scales, gallium particles can be directly internalized by macrophage immune cells, influencing associated physiological processes. When macrophages encounter lipopolysaccharide (LPS)—a component of the membrane of Gram-negative bacteria—they release NO and pro-inflammatory cytokines to eliminate pathogens. However, an overproduction of NO can non-specifically harm healthy tissues. Gallium nanodroplets (GNDs) can selectively curtail NO production by impeding the mRNA translation of iNOS proteins, thereby preserving Fe homeostasis.

## PERSPECTIVE

The diverse composition of living organisms, including the presence of flowing fluids such as blood, rigid skeletal structures, and soft tissues like muscles and fat, contributes to the dynamic nature of life in the natural world. Among various materials, LM biomaterials exhibit remarkable similarities to biological entities, existing in liquid, solid, and adjustable mechanical strength states. Consequently, LM holds great promise for manufacturing artificial organs, potentially advancing the development of cyborg-like entities. As a class of intelligent materials with rich physical and chemical properties, LM shows unique advantages in sensing, intelligent response, actuation, and so on. When designed as artificial organs, such as artificial electronic skin, artificial muscle, and electronic blood vessels, LM can achieve the enhancement of human capacity. Currently, research related to electronic skin has integrated EGaIn particles into crosslinked networks with protein molecules via metal-ligand coordinative interactions, yielding a biocompatible electronic skin with mechanical properties and self-healing capability [97]. In addition, Cheng *et al*. fabricated flexible and biodegradable circuits using LM wrapped by poly(L-lactide-co-ε-caprolactone), which became an electronic vessel by binding to triple vascular cells [98]. In future endeavors, LM can be employed to construct various cyborg tissues/organs to support or supplant human tissues and organs in executing their physiological functions. Taking the heart as an example, it is a vital organ responsible for circulating nutrient-rich blood to sustain bodily functions. Many devices have been developed to aid the heart in proper functioning. Cardiac pacemakers are medical devices that maintain or restore normal heart rhythm through electrical stimulation. These devices capture and analyze electrical signals, detecting abnormalities and providing necessary stimulation for proper cardiac performance. However, severe heart failure may require mechanical assistance alongside electrical stimulation to replace the pumping function of the heart. Currently, ventricular assist devices (VADs) are commonly employed in clinical practice to facilitate blood circulation by drawing blood from the ventricle and delivering it to the aorta or pulmonary artery. LM can be applied to assist the heart in its pumping function. For example, the inherent flexibility of LM enables the pumping system to effectively accommodate the dynamic movement of the heart, thereby minimizing the risk of mechanical injury to surrounding tissues. Furthermore, the conductivity of LM enables external energy transmission, potentially eliminating the need for batteries in existing pacemakers and VADs, thus avoiding their limitations and potential harm. Even more ambitiously, in cases of severe heart damage, an entirely flexible artificial heart using LM could redefine disease treatment by replacing the entire organ.

**Figure 9.**
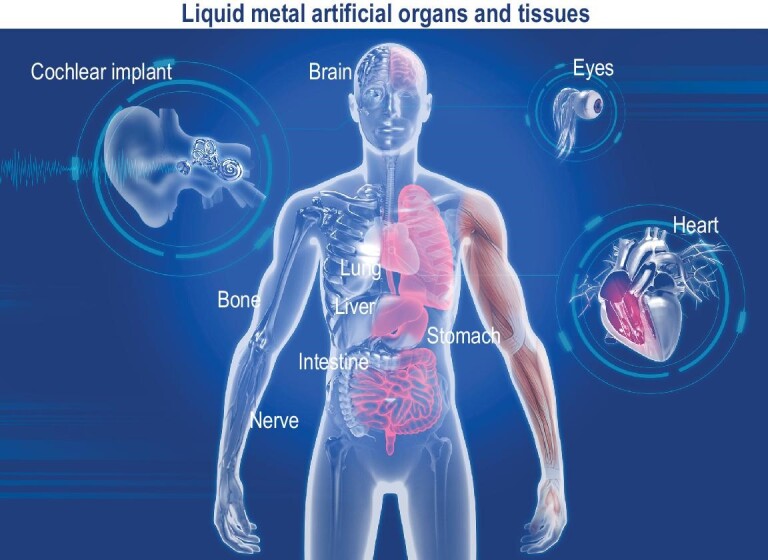
Imagination map of LM-enabled artificial organs, including the LM brain, cochlea, eyes, bones, nerves, lungs, heart, stomach, liver, intestines, pancreas, etc.

According to the aforementioned principles, LM-based tissue and organs like lungs, kidneys, intestines, pancreas, retinas, cochleas, and more can be developed. These could achieve tasks such as gas exchange, metabolic correction, monitoring gastrointestinal movements and microbiota, insulin and glucagon distribution, converting light signals to nerve signals for brain processing, and transforming acoustic signals to stimulate auditory nerves for hearing restoration (Fig. 9). These biomimetic tissues and organs aim to restore and potentially enhance various functions beyond natural human organs and tissues, advancing the field of LM-based regenerative medicine [99]. In the coming future, LM-based semi-bionic life entities are poised to emerge, ushering in the cyborg era with extraordinary physical capabilities through LM artificial organs, ultimately propelling the concept of LM artificial organs towards reality.

## CONCLUSION AND OUTLOOK

In summary, LM shows great potential for future clinical applications due to its biocompatibility, flexibility, electrical conductivity, and mechanical properties. This has led to its exploration in areas of medical imaging, wearable electronics, tissue engineering, and tumor therapy. Table 1 summarizes the applications of LM biomaterials in clinical areas mentioned above and compares them with existing clinical techniques.

**Table 1.** Summary of clinical applications, clinical challenges, LM characteristics and improved effectiveness of LM biomaterials.

Clinical applications	Clinical challenges	LM characteristics	LM advantages
Healthcare monitoring devices	Bulkiness, discontinuity and imprecision	Conductivity, softness	Real-time, continuity, precision
Eye trackers	Poor comfort and low precision	Conductivity, softness	Comfort, high precision
Intravascular contrast agents	Low signal-to-noise ratio	High density, fluidity	Clear-visibility of capillaries
External fixators	Complicated operation, not environmental protection	Low melting point	Easy operation, reusability
Bone cement	Complicated operation, displacement	Fluidity	Injectability
Exoskeleton	Restricted freedom	Low melting point	Rigidity and softness
Nerve interface	Nerve rupture, functional impairment	Conductivity	Restoration of electrical conduction
Deep brain stimulation	Stiffness	Conductivity, softness	Stiff-variable probe
Vascular embolic agent	Incomplete embolism, displacement	Fluidity	Complete embolism
Drug delivery	Poor effectiveness	Ligand-functionalized surface	Controlled release, collaborative therapy
Hyperthermia/cryoablation	Poor accuracy	Thermal/electrical conductivity	Precise therapy
Antibacterial and anti-inflammatory	Drug resistance	Unique chemical structure	New antimicrobial mechanism

For the coming LM translational medicine, current challenges are discussed below about LM biomaterials from laboratory advancements towards clinical translation to inspire every scientist and clinician considering:

1. Clinical application scenarios: Engineers should collaborate closely with frontline clinicians to discern the paramount challenges in disease treatment. This collaboration ensures that laboratory research outcomes possess relevance and potential for clinical translation.
2. Biosafety: Comprehensive *in vitro* cytotoxicity tests and *in vivo* biocompatibility assessments generally indicate that Ga, In, Bi, and inorganic Sn compounds exhibit negligible toxicity in moderate amounts, while organic Sn compounds manifest toxic properties. The toxicity of LM also depends on their presentation, predominantly as bulk LM or LM nanoparticles produced through sonication. Although LM nanoparticles can permeate cells and are often perceived to be more toxic than bulk LM, certain studies suggest that these nanoparticles degrade progressively within tumor environments, mitigating potential biotoxicity concerns associated with inorganic nanoparticles. Clinically, the FDA has sanctioned potassium nitrate for therapeutic purposes. Broadly, both bulk LM and LM nanoparticles are deemed to be biocompatible. Yet, current evaluations of LM toxicity predominantly rely on methods like cellular assays, blood analyses, and animal models and detailed mechanistic studies remain scarce. Future research in this domain is thus essential. Furthermore, the enduring *in vivo* safety and metabolism of bulk LM warrant thorough investigation to expedite the clinical translation of LM-based products [100].
3. Material stability: Owing to the unique physicochemical properties of LM and the inherent variability of the human body's internal milieu, rigorous testing is essential to verify product stability under diverse physiological circumstances and to develop appropriate encapsulation materials and methods that enhance stability.
4. Manufacturing technology: The complex anatomy of the human body demands unparalleled precision in LM products. Therefore, advancements in manufacturing technologies are imperative to guarantee the standardization and stability of materials throughout prolonged production.
5. Ethics: Beyond the established considerations tied to invasive experiments, the distinct attributes of LM introduce heightened ethical implications. Given humans’ innate capacity for independent thought, external elements that potentially manipulate their ability to process and relay information might infringe upon their autonomy, thereby testing existing ethical norms. Thus, the clinical application of LM requires not only rigorous standards for biosafety, material performance, preparation methodologies, and product functionality but also a deep reflection on ethical dimensions grounded in moral philosophy.

In conclusion, the clinical applications of LM reside at the interdisciplinary intersection of materials science, medicine, and biology. As research in these domains progresses, LM will continually innovate medical diagnostic technology and therapeutic approaches, paving the way for the development of the medical field. This expansion of the boundaries of contemporary medicine will empower humanity to tackle diverse medical challenges, carrying substantial implications for advancing clinical medicine, safeguarding human health, and enhancing living standards in the future.
